# The role of animals in the epidemiology and pathogenesis of severe fever with thrombocytopenia syndrome (SFTS): a scoping review

**DOI:** 10.1186/s40249-026-01505-x

**Published:** 2026-11-02

**Authors:** Jian Li, Zhebin Hu, Hai Tong, Saira Mustehsan, Muhammad Uzair Mukhtar, Nazrullozoda Sulaimon, Eman E. El Shanawany, Chimedtseren Bayasgalan, Zengyun Hu, Lei Duan, Zhaoyu Guo, Muxin Chen, Wei Hu, Xiaonong Zhou, Xinyu Feng

**Affiliations:** 1 Basic Medical College, Guangxi University of Chinese Medicine, Nanning, 530001 China; 2 School of Life Sciences, Inner Mongolia University, Hohhot, China; 3 School of Global Health, Chinese Center for Tropical Diseases Research, Shanghai Jiao Tong University School of Medicine, Shanghai, 20025 China; 4 School of Public Health, Shanghai Jiao Tong University School of Medicine, Shanghai, 20020 China; 5 Department of Medical Entomology and Parasitology, Institute of Public Health, Lahore, 54000 Pakistan; 6 Institute of Veterinary Medicine of the Tajik Academy of Agricultural Sciences, Dushanbe, Republic of Tajikistan; 7 Department of Parasitology and Animal Diseases, National Research Centre, El Buhouth St., Dokki, Cairo, Egypt; 8 Department of Infectious Diseases and Microbiology, School of Veterinary Medicine, Mongolian University of Life Sciences, Zaisan, Ulaanbaatar, 17024 Mongolia; 9 National Institute of Parasitic Diseases, Chinese Center for Disease Control and Prevention (Chinese Center for Tropical Diseases Research), NHC Key Laboratory of Parasite and Vector Biology, WHO Collaborating Centre for Tropical Diseases, National Center for International Research on Tropical Diseases, Shanghai, China; 10 Big Data Institute, Nuffield Department of Population Health, University of Oxford, Oxford, OX37LF UK

**Keywords:** Severe fever with thrombocytopenia syndrome, Animals, Epidemiology, One Health

## Abstract

**Background:**

Severe fever with thrombocytopenia syndrome (SFTS) is an emerging tick-borne zoonosis with a high case-fatality rate and expanding geographic range. Its persistence depends on complex cycles involving ticks and vertebrate animal hosts. A comprehensive understanding of animal roles is critical for predicting spillover risk and developing effective interventions under a One Health framework. This scoping review aimed to map and synthesize existing evidence on the roles of domestic animals, wildlife, and companion animals in the epidemiology, pathogenesis, and transmission of SFTSV.

**Methods:**

A scoping review was conducted following PRISMA-ScR guidelines. Multiple databases (MEDLINE, PubMed, Web of Science, China National Knowledge Infrastructure, Wanfang) and grey literature sources were searched from January 1, 2011 to October 21, 2025. Eligible studies involved non-human animals or ticks and confirmed SFTSV infection or exposure. Evidence was narratively synthesized across prespecified themes: animal host roles, clinical manifestations, transmission dynamics, and prevention strategies. We mapped the distribution of evidence across animal species, study designs, geographic regions, diagnostic approaches, and thematic domains.

**Results:**

The review identified a broad animal host range comprising at least 15 vertebrate species across domestic, wildlife, and companion categories. Domestic ruminants show high seroprevalence and act as primary amplification hosts. Wildlife (e.g., rodents, shrews) contribute to enzootic maintenance. Cats are highly susceptible, develop severe disease with high viral loads, and are a major source of direct non-vector transmission to humans. Dogs often have mild illness but can shed virus persistently. Transmission primarily occurs via the tick *Haemaphysalis longicornis*, but direct transmission via contact with blood/secretions of sick animals (especially cats and farmed mink) is significant. Prevention requires integrated tick management, biosecurity in veterinary/farm settings, and public education.

**Conclusions:**

Animals are central to the ecology of SFTSV. Current evidence underscores the necessity of integrated, multisectoral One Health strategies for surveillance, risk communication, and control. Future research priorities include standardizing host-competence studies, harmonizing diagnostics, elucidating comparative immunopathology, and evaluating the feasibility and impact of intervention bundles, including potential veterinary vaccines.

**Supplementary Information:**

The online version contains supplementary material available at https://doi.org/10.1186/s40249-026-01505-x.

## Background

Severe fever with thrombocytopenia syndrome (SFTS) is an emerging tick-borne zoonosis caused by SFTS virus (SFTSV; genus *Bandavirus*, family *Phenuiviridae*). Since its initial description in China in 2009, SFTS has been reported across East Asia, including Japan and the Republic of Korea, with more recent detections in Southeast Asia, underscoring a widening geographic footprint and growing public health concern [1–4]. Human disease is characterized by acute febrile illness, thrombocytopenia, leukocytopenia, and gastrointestinal symptoms, with a proportion of patients progressing to multiorgan dysfunction and hemorrhagic manifestations; reported case-fatality ratios range from 5 to 30% and are highest among older adults and those with comorbid conditions [5–7]. Therefore, elucidating the maintenance and transmission mechanisms of SFTSV in nature, particularly the critical roles played by animal hosts, is essential for a comprehensive understanding of the disease's epidemiology.

SFTSV persistence depends on complex enzootic cycles that link hematophagous ticks, most notably *Haemaphysalis longicornis*, with diverse vertebrate hosts, which vary in their exposure patterns, viremia kinetics, and shedding profiles [8–10]. Beyond vector-borne pathways, accumulating evidence implicates direct animal-to-human transmission in household, veterinary, and farm settings, especially from ill cats and, in some reports, dogs and fur animals, broadening the risk landscape beyond tick bites alone [11–15]. Understanding animal roles on epidemiology and pathogenesis is therefore central to predicting spillover risk, prioritizing prevention, and designing countermeasures under a One Health framework.

This scoping review synthesized the state of knowledge on animals in SFTSV epidemiology and pathogenesis. We (i) assessed domestic animals, wildlife, and companion animals as reservoirs, amplifiers, and spillover interfaces; (ii) summarized clinical manifestations across species and their mechanistic correlates; (iii) integrated transmission evidence spanning ticks, animals, and humans, including experimental studies; and (iv) evaluated prevention and control strategies, therapeutics and vaccines, and the fitness-for-purpose of animal models. We conclude with research priorities aimed at strengthening evidence and accelerating translation to practice.

## Methods

### Review design and objectives

This review was conducted as a scoping review to collect and synthesize the breadth of evidence on the roles of animals in the epidemiology and pathogenesis of SFTS across domestic, wildlife, and companion species, and at the animal–tick–human interface. We followed the methodological framework for scoping reviews and reported the review in accordance with the PRISMA extension for Scoping Reviews (PRISMA-ScR) 2018 guidance [16].

### Information sources and search strategy

We searched MEDLINE via PubMed and Chinese bibliographic databases, including CNKI and WanFang, from January 1, 2011 to October 21, 2025. The start date was selected because SFTS was first recognized as a distinct emerging infectious disease in China around this period. Grey literature was searched to capture surveillance reports, technical guidance, outbreak reports, and policy documents relevant to animal infection, animal-associated transmission, or One Health prevention. Sources included WHO, WOAH/OIE, FAO, national or regional CDCs, and reference lists of relevant reviews and included articles. The search strategy combined controlled vocabulary and free-text terms related to the disease, virus, vectors, and animal hosts. Search blocks included terms for SFTS or SFTSV, tick vectors, and animal host categories. Detailed search strategy and study selection are provided in Supplementary Table 1.

### Eligibility criteria

We considered studies involving non-human animals (including livestock and companion animals, and wild animals) and ticks, as well as reports of cross-species transmission that involved human cases. Eligible studies needed to confirm SFTSV infection or exposure through methods such as virus isolation, nucleic acid amplification (e.g., PCR, Polymerase Chain Reaction), antigen detection, or serology. Outcomes of interest encompassed infection and seroprevalence, viremia kinetics and viral shedding, clinical signs and pathological findings, vector competence and virus maintenance, direct and indirect transmission pathways, and the performance of diagnostic assays or intervention strategies. A broad range of study designs was included, such as field surveys, case reports and series, observational analytical studies, experimental infections, vector competence assays, outbreak investigations, and relevant reviews for citation mining. Studies were excluded if they focused on non-SFTSV pathogens, were purely in vitro experiments without direct in vivo or field relevance, presented opinion pieces without primary data, employed assays with unclear targets, or represented duplicate datasets.

### Study selection and synthesis

All identified records were imported into a reference management database (EndNote X21), and duplicates were removed before screening. The screening was conducted in two phases: first, titles and abstracts were screened against the eligibility criteria, and subsequently, the full texts of potentially relevant records were retrieved and assessed for final inclusion. This process was performed independently by two reviewers, with any discrepancies resolved through discussion or consultation with a third reviewer. Given the scoping review design aimed at mapping the evidence landscape, the synthesis of findings was narrative and thematic rather than quantitative. The extracted data were charted and synthesized to describe the key concepts, evidence patterns, and gaps in the literature regarding animal roles in SFTSV epidemiology and pathogenesis, as structured around the review's prespecified objectives within the One Health framework.

### Definitions of wildlife categories and classification of animal host roles

Following Goulet et al. [17], we distinguished free-roaming wildlife, peri-domestic wildlife, and farmed or captive wildlife to avoid overgeneralizing evidence across ecologically distinct animal groups. Free-roaming wildlife was defined as non-domesticated animals living independently in natural or semi-natural ecosystems without routine human provisioning or confinement. Peri-domestic wildlife referred to non-domesticated or commensal species living near human settlements, farms, or livestock facilities and potentially bridging sylvatic and domestic interfaces. Farmed or captive wildlife referred to non-domesticated species maintained under human management or commercial production systems, such as mink or foxes.

Because terms such as reservoir, amplifier, sentinel, and spillover host have specific ecological meanings, we defined them a priori for this review. A reservoir was defined as a host or host community capable of contributing to the long-term maintenance of SFTSV in nature. An amplifier was defined as a host in which infection may generate sufficient viremia or shedding to increase transmission probability to ticks, humans, or other animals. A sentinel was defined as a species whose infection or seropositivity indicates local SFTSV circulation but does not necessarily imply a major role in onward transmission. A spillover host was defined as a host infected through contact with the enzootic cycle but not clearly required for viral maintenance.

### Synthesis of evidence

Given the scoping review design and heterogeneity of included evidence, findings were synthesized narratively and thematically rather than quantitatively. We did not conduct meta-analysis or formal risk-of-bias grading. Instead, we mapped the distribution of evidence across animal species, study designs, geographic regions, diagnostic approaches, and thematic domains.

The synthesis was structured around four prespecified themes: animal host roles, clinical manifestations and pathogenesis in animals, transmission pathways, and One Health prevention and control. Within each theme, we distinguished well-supported evidence from plausible but unconfirmed ecological inference. Where evidence was limited, inconsistent, or based mainly on serology or single reports, this uncertainty was explicitly stated.

## Results

### Search findings

**Fig. 1:**
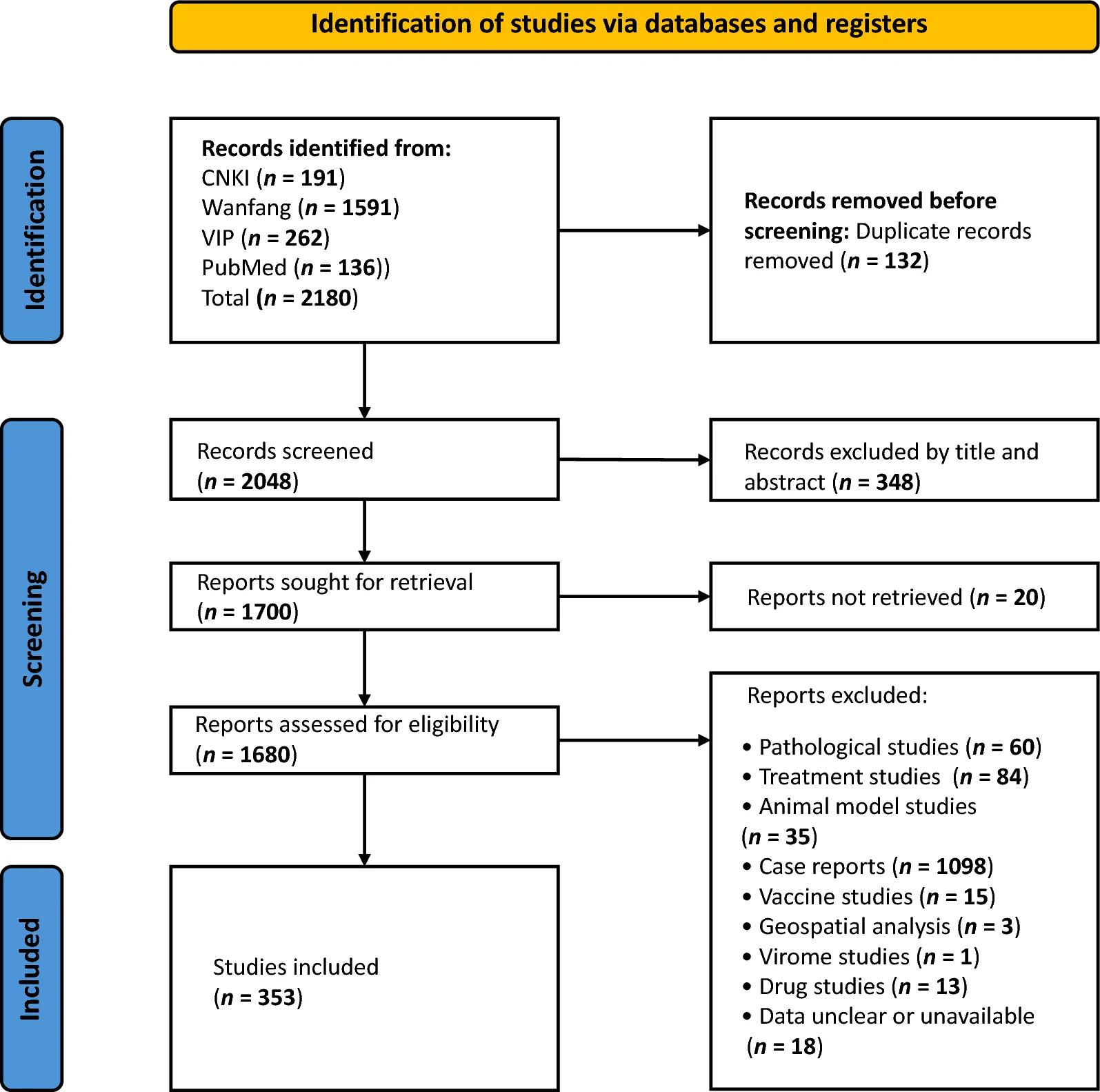
PRISMA flow diagram of study selection for the scoping review on the role of animals in the epidemiology and pathogenesis of severe fever with thrombocytopenia syndrome. *CNKI* China National Knowledge Infrastructure, *WanFang* Wanfang Data, *VIP* Database for Chinese Technical Periodicals

Across four sources (CNKI, WanFang, VIP, and PubMed), we identified 2180 records (CNKI *n* = 191; WanFang *n* = 1591; VIP *n* = 262; PubMed *n* = 136). After removing 132 duplicates, 2,048 unique records were screened by title/abstract, and 348 were excluded at this stage. We sought retrieval of 1,700 full-text reports; 20 could not be retrieved, leaving 1,680 reports assessed for eligibility. Following full-text assessment, 353 studies met the inclusion criteria and were included in the scoping review. The most common reasons for full-text exclusion were case reports (*n* = 1098), treatment studies (*n* = 84), pathological studies (*n* = 60), and animal model studies (*n* = 35), with additional exclusions due to vaccine studies (*n* = 15), drug studies (*n*= 13), geospatial analyses (*n* = 3), virome studies (*n* = 1), and unclear data or insufficient sample size (*n* = 18) (Fig. 1).

To improve transparency of the evidence base, we summarized all included studies in Supplementary Table S2. For each study, we extracted publication year, country or region, study design, animal or tick species investigated, diagnostic approach, language, and primary thematic focus. The included studies spanned field serosurveys, molecular detection studies, outbreak investigations, experimental infection studies, vector-related studies, animal case reports or case series, and reviews used for citation tracking.

### Animal hosts: reservoirs, amplifiers, and spillover interfaces

The persistence and spread of SFTSV in nature are dependent on a complex enzootic cycle involving ticks and vertebrate hosts. Animals serve as the primary reservoirs and amplification hosts, where the virus can replicate to levels sufficient to infect feeding ticks, thereby maintaining the transmission cycle [18]. Extensive epidemiological investigations have revealed that SFTSV has an exceptionally broad host range, infecting a diverse array of both domestic, wild and companion animals. The grading of animal host roles in SFTSV ecology is summarized in Table 1.

**Table 1:** Grading of animal host roles in severe fever with thrombocytopenia syndrome virus ecology

Host group	Role classification	Certainty (qualitative)	Key evidence pillars	References
*Domestic animals*				
Domestic ruminants (goats, sheep, cattle, camel)	Amplifier (primary); Sentinel (context-dependent)	Moderate–High	∙ Field serology; ∙ Polymerase chain reaction (PCR) ∙ Occasional isolation; ∙ Tick acquisition from ruminant viremia; ∙ Associations with husbandry and grazing	[19, 20, 22, 24–29]
*Wildlife (free-roaming, peri-domestic, and farmed or captive)*				
Free-roaming, peri-domestic animals (shrews, hedgehogs, wild boar, rodents)	Reservoir/Amplifier (landscape-dependent)	Moderate	∙ Serology/PCR; ∙ Hosts for immature *Haemaphysalis*; ∙ Spatial-seasonal concordance with tick infection	[8–10, 22, 30]
Fur animals (mink, fox)	Amplifier (occupational interface)	Low–Moderate	∙ Outbreak ∙ Cluster investigations; ∙ Aerosolization during skinning or handling plausible	[31–34]
*Companion animals*				
Dogs	Sentinel/Amplifier (secondary)	Low–Moderate	∙ Serology; ∙ Occasional PCR; ∙ Prolonged urinary shedding; Generally low clinical severity	[14, 20–22, 35]
Cats	Amplifier & direct spillover source	Moderate–High	∙ High viral loads; ∙ Severe disease; ∙ Multiple shedding routes; ∙ Clusters implicating cat-to-human transmission	[11–15, 23, 36, 37]

#### Domestic animals

Serological surveys consistently report high exposure among small ruminants and cattle, with moderate and lower levels exposure in pigs and chickens[18–22]. A meta-analysis estimated pooled seroprevalence at 45.7% in goats/sheep, and 36.7% in cattle [20]. Although PCR positivity is far lower, consistent with brief viremia, detection of viral RNA and occasional virus isolation in livestock confirm active infection [19–23]. Free-range or pasture access and tick abundance correlate with higher seropositivity, implicating environmental exposure as a dominant driver [20]. Village-level investigations in endemic areas have found higher SFTSV RNA prevalence in domestic animals than in humans or questing ticks, underscoring animals' central role in local maintenance and spillover interfaces [24].

#### Wildlife

Wildlife-related evidence was interpreted according to ecological context, distinguishing free-roaming wildlife, peridomestic species, and farmed or captive wildlife. Among free-ranging wildlife, SFTSV infection or exposure has been documented in wild boar, small rodents, shrews, and hedgehogs, with geographic variation in the species detected [8–10, 22, 30]. These findings suggest a potential role for wildlife in sylvatic maintenance; however, most evidence derives from serology or viral RNA detection, and species-specific competence, viremia duration, and capacity to infect feeding ticks remain insufficiently defined. Small mammals may be especially relevant because they serve as hosts for immature *Haemaphysalis* stages and could link wildlife communities with local tick maintenance cycles [8–10]. Peri-domestic wildlife and commensal small mammals may further bridge sylvatic, livestock, and human-associated environments, although direct evidence for independent transmission cycles remains limited. In contrast, farmed or captive fur animals, such as mink, represent a distinct high-density, human-managed interface. Isolation and identification of SFTSV from fur-animals expand the spectrum of susceptible animal species and highlight occupational exposure risks during animal handling or contact with blood, secretions, and tissues [31, 38]. These findings should be interpreted separately from free-roaming wildlife because they reflect managed production settings rather than natural sylvatic circulation.

#### Companion animals

Cats and dogs demonstrate frequent exposure in endemic regions, yet clinical trajectories diverge markedly by species. Cats are highly susceptible, with severe or fatal disease documented in field and experimental settings [13, 36], and high viral loads in blood and multiple secretions/excretions [14, 23, 37]. Clusters and case reports implicate contact with ill pets, especially cats, as a source of human infection [11, 12, 15]. Dogs are often asymptomatic or mildly ill but may shed the virus in their urine for prolonged periods, thereby extending the potential zoonotic risk [14, 35]. Additionally, the occupational risk for veterinarians and veterinary nurses has been highlighted by Kirino et al., who found seroprevalence of SFTSV antibodies among small-animal practice workers in Japan [15]. These findings highlight the importance of considering companion animals as potential sources of SFTSV transmission and the need for veterinary professionals to be aware of the disease.

### Clinical manifestations and outcomes in animals

Most livestock infections are subclinical or mild, aligning with their ecological roles rather than overt disease [19–23]. In striking contrast, cats can develop fulminant viral hemorrhagic fever, with fever, anorexia, cytopenias, hemorrhage, and high lethality; pathology often reveals necrotizing lymphadenitis and hemophagocytosis, paralleling fatal human cases [13, 36, 39]. Dogs may present with fever and cytopenias but generally recover with supportive care, though prolonged shedding can persist beyond clinical resolution [14, 35]. Direct observation of clinical signs of SFTS in free-ranging wildlife is exceptionally rare due to the challenges of monitoring these populations. These species gradients suggest differences in innate immune restriction and tissue tropism. Cytokine dysregulation, as documented in severe human SFTS, is characterized by elevated IFN-α/γ, IL-6, IL-10, and G-CSF, as well as IP-10. This provides a mechanistic framework relevant to severe feline disease and informs model selection for pathogenesis and therapeutic evaluation [40–42].

### Transmission dynamics

#### Tick vectors

**Fig. 2:**
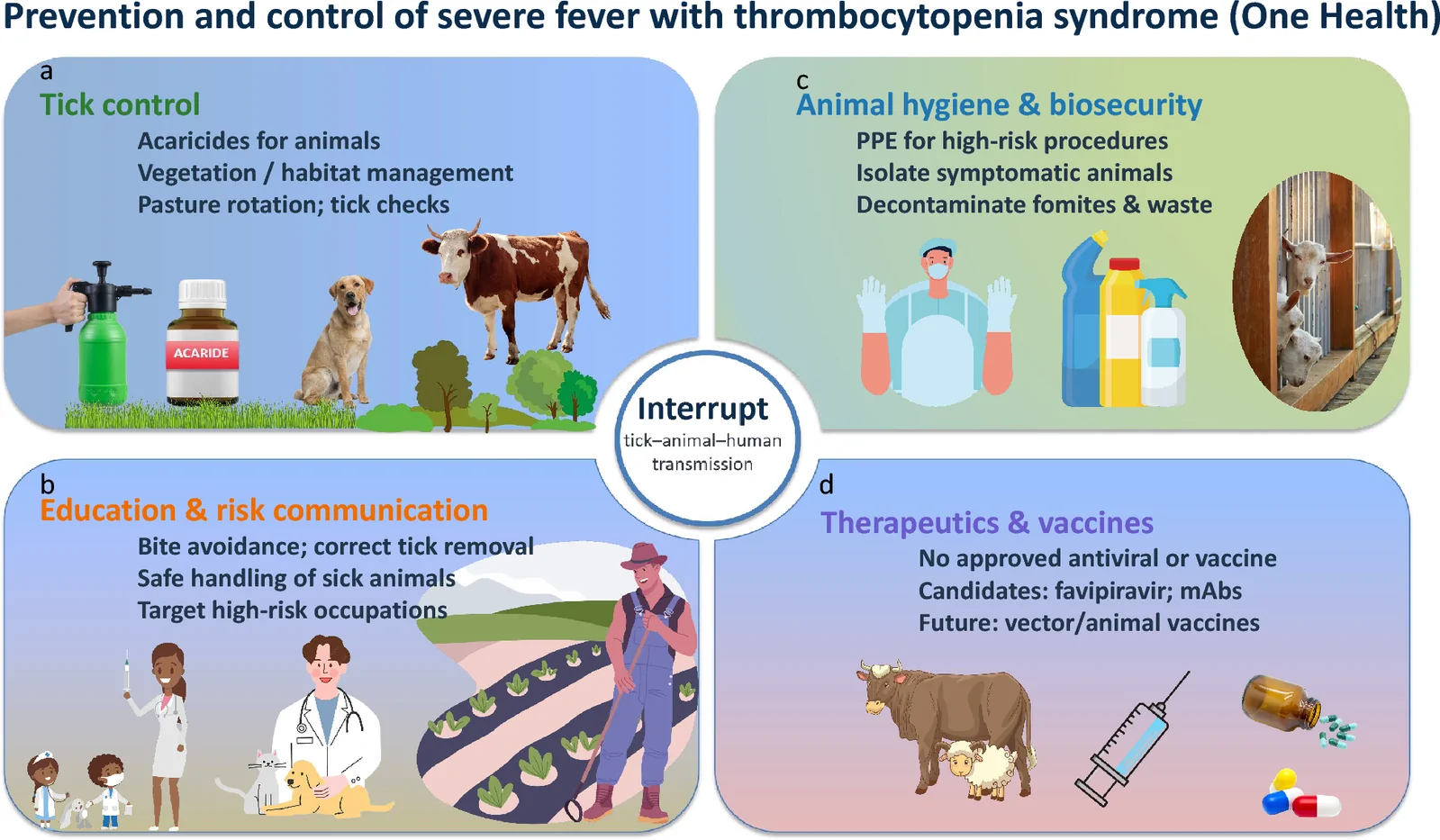
Schematic of severe fever with thrombocytopenia syndrome transmission interfaces among wildlife, domestic, companion animals and humans

Evidence suggests that SFTSV transmission between animals primarily occurs through tick vectors (Fig. 2). *Haemaphysalis longicornis* is the principal vector, with field and experimental data demonstrating acquisition from viremic hosts and transstadial/transovarial maintenance; detection of SFTSV in other tick species, like *Haemaphysalis flava*, *Ixodes sinensis*, and *Rhipicephalus sanguineus,* suggests multi-vector potential in some ecologies [1, 5, 18, 21, 43, 44]. Vector competence varies by tick species and stage, virus strain, and environmental conditions, and is modulated by host community composition.

The central tick represents *Haemaphysalis longicornis*, the principal recognized vector of SFTSV. The figure illustrates major ecological interfaces rather than assigning specific tick life stages to specific host groups. Solid arrows indicate well-supported vector-mediated transmission pathways involving ticks and vertebrate hosts.

#### Animal-to-human

Beyond tick bites, direct transmission to humans has been inferred from epidemiological investigations, genomic linkage, and virological data, most convincingly from ill cats, dogs and mink [11–15, 23, 31, 38]. In one instance in Japan, a woman died of SFTS after being bitten by a cat with no visible tick bites, suggesting cat-to-human transmission [1]. A similar case report exists of a companion dog contracting SFTS and displaying clinical signs [35]. High viral loads in feline tissues and secretions, and prolonged canine urinary shedding, provide biological plausibility for exposure via bites, scratches, aerosols during intensive handling, or contact with contaminated environments [13, 14, 23].

A recent investigation in Weihai, China, explored a cluster of two SFTS cases among workers on a mink farm [31]. Genetic analysis revealed that the viral sequences from the patients, the farm animals, and environmental samples were 99.9% homologous. It was suspected that the workers were directly infected from the animals, possibly through aerosol transmission generated during the skinning process or via direct contact with blood and bodily fluids [31]. This report expands the scope of potential animal-to-human transmission to include occupational exposures on farms, highlighting another facet of the complex epidemiology of SFTS.

#### Animal-to-animal

Transmission between animals is limited but plausible. In a ferret model, SFTSV was shed in large amounts through nasal discharge, saliva, and urine for up to 12 days post-infection [45]. Subsequent experiments showed that the virus could be transmitted from infected to naive ferrets through both direct and indirect close contact, particularly via exposure to contaminated serum and urine [46]. In companion animals, close contact, high titers, and multiple shedding routes may permit sporadic animal-to-animal transmission among cats, although field confirmation remains limited [13, 14, 23, 36]. In contrast, experimental co-housing of infected and naive goats without ticks has not demonstrated transmission, implicating ticks as required mediators in typical ruminant husbandry settings [47]. However, the increasing reports of direct animal-to-human transmission, often through contact with the blood or body fluids support the biological plausibility of direct animal-to-animal spread.

#### Human-to-human

Human-to-human transmission is uncommon but has been documented, particularly through direct contact with the blood or body fluids of infected individuals [48–50]. Such transmission events are often observed in specific settings, leading to familial and nosocomial clusters. For example, several reports have detailed family clusters where secondary cases developed symptoms after close contact with an index patient. Healthcare settings have also been sites of SFTSV transmission [48]. One notable report identified a family cluster where transmission was linked to potential ocular exposure to infectious blood [51]. The potential for human-to-human to cause both symptomatic and asymptomatic infections highlights the need for robust surveillance and stringent infection control practices.

#### Human-to-animal

The focus of public health and research has been on preventing zoonotic spillover and subsequent human-to-human spread. However, the direction of transmission in the available literature is consistently from the enzootic cycle (ticks and animals) to humans. There is currently no scientific evidence in the provided references to support the transmission of SFTSV from humans to animals. While theoretically possible, this transmission pathway remains uninvestigated and unconfirmed.

#### Experimental and molecular confirmation

Laboratory studies have provided direct evidence for the role of animals in the SFTSV transmission cycle. Experiments have demonstrated that SFTSV can be efficiently transmitted between ticks and laboratory-bred mouse strains. Ticks fed on SFTSV-infected mouse successfully acquired the virus, which was then transmitted both transstadially and transovarially. Subsequently, these infected ticks were able to transmit the virus to naive mouse during feeding [43]. These experiments provide experimental support for the biological plausibility of a tick–rodent–tick transmission cycle. However, they should not be interpreted as confirmatory evidence that free-ranging or peridomestic rodents serve as amplification hosts in natural SFTSV transmission. Field-based studies integrating rodent infection status, viremia kinetics, tick infestation, and viral genetic linkage are needed to establish whether peridomestic rodents contribute to sylvatic or peri-domestic maintenance cycles. In another study, experimentally infected goats did not transmit the virus to co-housed naive goats, indicating that direct transmission between these animals is unlikely. However, when naive sentinel goats were placed in an endemic field environment, they became infected shortly after tick infestation, confirming that ticks are the essential vector for transmission among these hosts [47]. Second, molecular evidence showed that SFTSV strains isolated from a naturally infected goat’s serum and from the ticks feeding on it shared 100% S‑segment sequence homology, directly confirming viral acquisition from goats by ticks. Together, these findings underscore the central role of a wide range of animal species in sustaining SFTSV in nature, primarily through a tick-vectored cycle.

### Prevention and control strategies (One Health)

#### Tick control

**Fig. 3:**
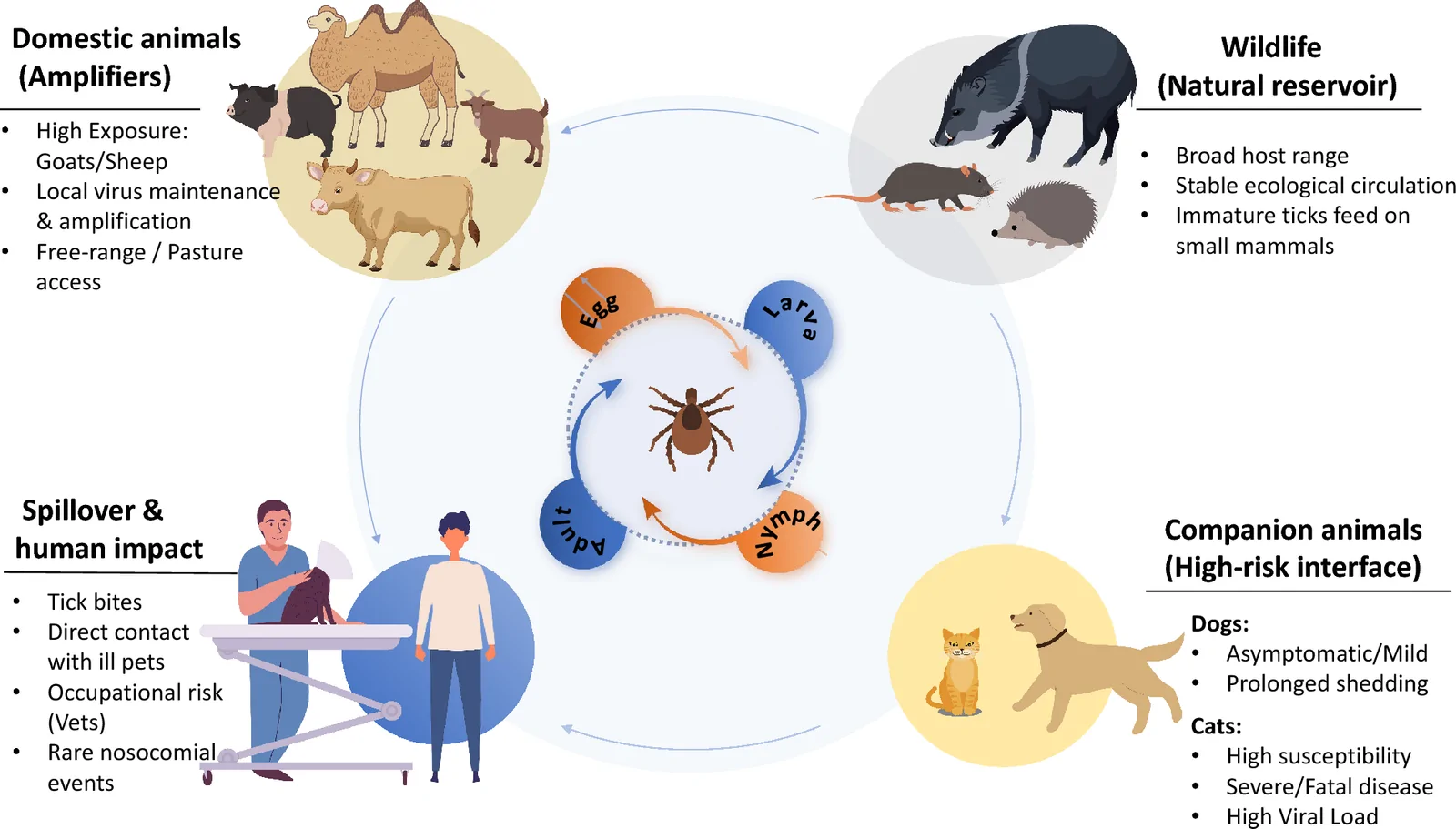
One Health strategies for the prevention and control of Severe Fever with Thrombocytopenia Syndrome (SFTS). **a** Integrated tick management, combining chemical control, environmental modification, and personal protection; **b** Animal hygiene and environmental management, emphasizing biosecurity, isolation of sick animals, and husbandry practices to reduce tick exposure; **c** Targeted public and veterinary health education for high-risk groups; and **d** Development of therapeutics, human and veterinary vaccines, and anti-tick vaccines. *SFTS* severe fever with thrombocytopenia syndrome, *PPE* personal protective equipment, *mAbs* monoclonal antibodies

Integrated tick management remains the cornerstone of control strategies [52]. Given the broad host range of *Haemaphysalis longicornis* and its ability to reproduce via parthenogenesis in certain regions, making populations particularly difficult to eradicate, the implementation of multi-pronged and sustained integrated control measures is crucial. Core strategies encompass: chemical control, such as routine acaricide applications for livestock and companion animals; environmental management, including vegetation modification, fencing, and pasture rotation to reduce tick habitats and host exposure risk; and personal protection, emphasizing regular tick checks and prompt removal of attached ticks [53]. These measures are mutually reinforcing, collectively forming an effective barrier aimed at reducing environmental tick densities, thereby blocking the transmission cycle of SFTSV among animals, ticks, and humans (Fig. 3a).

#### Animal hygiene and environment management

Robust biosecurity protocols on farms and in veterinary clinics are critical for mitigating direct transmission risks at the animal-human interface. Foundational measures include the consistent utilization of appropriate personal protective equipment (e.g., gloves, masks, eye protection) during high-risk procedures such as the handling of sick or febrile animals; the prompt isolation of symptomatic animals; and rigorous decontamination of contaminated equipment (fomites) and animal waste [7, 15] (Fig. 3b). Complementing these, proactive husbandry and management practices designed to minimize tick exposure are strongly advised in endemic areas. These practices, which include controlled grazing schedules, regular mowing of pasture perimeters, and the installation of physical barriers, serve to reduce host-vector contact, thereby functioning as a primary line of defense alongside direct biosecurity measures [20, 44].

#### Public-health and veterinary education

Effective public health education serves as a critical component of SFTS prevention, with risk communication strategies needing to clearly convey key protective behaviors. These include bite avoidance through personal protective measures, the correct technique for tick removal to reduce backflow inoculation, safe handling practices when dealing with sick pets or livestock, and the imperative of early medical consultation following a febrile illness after potential exposure (Fig. 3c). This educational outreach must be particularly targeted, providing essential occupational guidance for high-risk groups such as veterinarians, veterinary nurses, animal groomers, and farm workers in endemic regions, who face amplified exposure risks in their professional duties [15, 23].

#### Therapeutics and vaccines

Currently, no specific antiviral therapy is approved for the treatment of SFTS in humans. Among investigational therapeutics, favipiravir has demonstrated promising efficacy in animal models and has progressed to clinical evaluation. Other candidates, such as monoclonal antibodies targeting the viral glycoprotein (Gn), have demonstrated protective potential in preclinical studies [2, 54, 55]. On the prevention front, although no licensed vaccine against SFTSV is yet available, diverse vaccine platforms, including live-attenuated, viral-vectored, and DNA-based candidates, have been developed and have shown encouraging immunogenicity in animal models. However, correlates of protection and the breadth of efficacy against circulating genetic variants remain to be clearly defined [56–61]. An alternative vaccination strategy that has shown promise targets the tick vector itself. Vaccines against tick bites, designed to disrupt the feeding process or pathogen transmission, represent a promising complementary approach by potentially reducing vector competence and burden. Furthermore, the future veterinary vaccination of key animal amplifiers, such as small ruminants or dogs, could serve as a strategic component to reduce viral circulation within the reservoir, contingent upon the demonstration of field effectiveness and cost-effectiveness [56–60] (Fig. 3d).

## Discussion

Animals are central to the ecology, persistence, and clinical impact of SFTSV, and their roles must be interpreted through a One Health lens that links vertebrate communities, vector dynamics, and human exposure. Across endemic landscapes, domestic ruminants and dogs sustain high levels of exposure and most plausibly operate as amplifiers or sentinels depending on management practices, tick pressure, and seasonality, whereas cats occupy a distinct niche as highly susceptible hosts capable of severe disease with high-titer shedding that bridges household and veterinary settings [13, 14, 19–24, 35, 36]. Wildlife communities contribute to ecological redundancy in enzootic maintenance, as small mammals hosting immature *Haemaphysalis* are likely to underpin seasonal oscillations and spatial heterogeneity in risk [8–10, 22, 30]. These cross-species patterns argue against siloed, human-only surveillance and instead support integrated systems that triangulate human case data with animal serology/PCR and tick infection metrics to identify foci, anticipate peaks, and trigger pre-emptive, area-specific interventions [10, 44, 62].

Furthermore, *c*omparative pathology clarifies why some species disproportionately contribute to human risk and provides mechanistic insights for targeted interventions. Specifically, the convergent immunopathology between severely affected cats and fatal human SFTS, including cytopenias, necrotizing lymphadenitis, hemophagocytosis, and cytokine-storm signatures, highlights shared pathogenic pathways (e.g., excessive inflammatory responses) that could be therapeutically targeted [13, 39–41]. By contrast, dogs commonly exhibit mild or subclinical illness, yet may shed the virus in their urine for prolonged periods, subtly extending the environmental contamination window [14, 35]. These interspecies contrasts imply differences in innate immune restriction, tissue tropism, and possibly host genetics that remain insufficiently mapped. Vector biology is another pivotal uncertainty: competence varies across tick species and stages, and mechanisms of vertical maintenance (transovarial, transstadial, and co‑feeding) and overwintering govern the durability of local transmission systems [1, 5, 18, 21, 43, 44]. Because vertebrate viremia profiles determine tick acquisition probabilities, precise quantification of peak titers and duration across species is not merely academic, it defines which hosts are true amplifiers and when they matter most.

Transmission is multifactorial. Vector-mediated cycles dominate at the population scale, but direct animal-to-human exposures are documented in households, clinics, and farms. Additionally, nosocomial events, though uncommon, pose a serious risk where contact with blood or body fluids is not adequately protected [11–15, 23, 31, 38, 63]. The most compelling evidence for non-vector spillover centers on cats, reflecting high viral loads in blood and multiple secretions/excretions, clinical severity that facilitates detection, and epidemiological clusters with plausible exposure windows for owners and veterinary staff [11–15, 23]. Reports of infection from fur-animal operations (e.g., mink) expand the at-risk domestic portfolio and suggest that aerosol generation or intensive handling (e.g., skinning) may amplify exposure, warranting heightened biosecurity [31, 38]. These realities expand prevention beyond “avoid tick bites” and demand a surveillance architecture that couples animal and tick data to human case surveillance. Practically, this entails multispecies sampling at the human–animal interface, harmonized diagnostic panels (PCR targets, neutralization assays, Ct/PRNT thresholds) across veterinary and human laboratories, and integrated analytics that fuse time-stamped animal seroconversion, tick infection rates, meteorological covariates, and human notifications to forecast risk and target interventions [10, 62].

Given the SFTSV infection data and research evidence we have presented, several low‑cost and readily feasible prevention and control measures should be prioritized. For example, appropriate PPE for pet owners and veterinary staff when handling febrile or clinically suspect cats in endemic areas; standardized clinic protocols for triage, isolation, and environmental decontamination; and stringent biosecurity during high-risk farm tasks that generate aerosols or involve contact with blood and offal [11, 12, 14, 15, 23, 31, 38]. In parallel, a maturing pipeline of antivirals and vaccine candidates should explicitly leverage cross-species models using fitness-for-purpose criteria—IFN-pathway-deficient mouse for early pathogenesis and antiviral screening; ferrets and cats for clinically relevant disease, shedding, and potential transmission questions; and targeted ruminant models for field-reflective exposure biology [2, 38, 54, 56–60]. Harmonized diagnostic standards are enabling infrastructure for both implementation and trials, providing comparable endpoints and facilitating the definition of correlates of protection. Finally, veterinary vaccination—directed at key amplifiers such as small ruminants or dogs, where epidemiology supports a meaningful contribution to environmental amplification—deserves rigorous evaluation for effectiveness and cost-effectiveness, and could complement human vaccination once available [56–60].

These scientific and operational insights crystallize an immediately actionable research agenda. First, standardized, longitudinal host-competence studies should quantify species-specific viremia trajectories relative to experimentally determined tick-infection thresholds, enabling definitive classification of reservoirs, amplifiers, and sentinels in different ecologies [2, 20, 43, 47, 56, 57, 60]. Second, given that systematic validation of current diagnostic methods across different vertebrate species remains a notable gap in the field, we propose that future research prioritizes the development of harmonized, multispecies diagnostic panels. Third, comparative immunology to explain species-specific clinical trajectories (cats vs dogs vs ruminants) should integrate cytokine profiling and controlled infection models to identify determinants of severe disease and therapeutic targets [13, 39–41]. Fourth, rigorous vector competence protocols with agreed-upon reporting standards, for example, dose calibration, feeding mode, blinding, replicate numbers, and contamination safeguards are needed to stabilize estimates across laboratories and environments [1, 5, 18, 21, 43]. Fifth, implementation research should evaluate One Health bundles (tick management, veterinary biosecurity, occupational PPE, and, where justified, veterinary vaccination), with attention to feasibility, acceptability, equity, and cost-effectiveness in resource-limited rural settings that carry disproportionate risk [10, 20, 47, 56, 57, 60, 62]. Collectively, these priorities aim to convert descriptive knowledge into quantitative, policy-ready evidence that is embedded in routine veterinary and public health practice.

In policy terms, guidance must extend beyond vector avoidance to encompass robust biosecurity in veterinary clinics and farms, explicit PPE recommendations for handling febrile pets, especially cats, and clear occupational standards for workers in high-exposure settings. Integrated surveillance linking human, animal, and vector data should be institutionalized so that risk communication and control actions are triggered by predefined thresholds rather than ad hoc judgments. Where local epidemiology supports it, pilot veterinary vaccination of key amplifiers, paired with human vaccination when available, may reduce environmental amplification and lower spillover risk, provided programs are formally evaluated and resourced (14, 15, 23, 31, 38, 56–60). Ultimately, reducing the human burden of SFTS depends on attenuating transmission pressure across the ecological network that sustains the virus. By aligning mechanistic understanding with pragmatic interventions, such as coherent surveillance, standardized diagnostics, fitness-for-purpose models, and accountable implementation, programs can transition from reactive containment to anticipatory control, yielding measurable and equitable benefits for both human and animal health.

## Conclusions

Animals contribute to SFTS ecology in heterogeneous ways, with stronger evidence for domestic ruminants and companion animals than for free-roaming wildlife, whose role in sylvatic maintenance remains plausible but unconfirmed by direct experiments. Effective prevention and control, therefore, necessitate an integrated One Health strategy that synergistically combines sustained vector control, stringent biosecurity protocols on farms and in veterinary clinics, and targeted risk communication for high-risk occupational groups. Concurrently, the accelerated development of effective vaccines and specific antiviral therapies remains a global priority. Ultimately, the implementation of coordinated, multi-sectoral surveillance and intervention packages represent the most pragmatic and promising path toward reducing the burgeoning burden of SFTS across its expanding endemic range.

## Supplementary Information

Supplementary Material 1. Table S1: The detailed search strategy in this study including all database retrieving procedure

Supplementary Material 2. Table S2: The final included studies in the role of animals in the epidemiology and pathogenesis of severe fever with thrombocytopenia syndrome: a scoping review

## Data Availability

No datasets were generated or analysed during the current study.

## Abbreviations

SFTS: Severe fever with thrombocytopenia syndrome
SFTSV: Severe fever with thrombocytopenia syndrome virus
CNKI: China National Knowledge Infrastructure
Ct: Cycle threshold
DNA: Deoxyribonucleic acid
FAO: Food and Agriculture Organization
G-CSF: Granulocyte-colony stimulating factor
Gn: Glycoprotein
IFN-α/γ: Interferon-alpha/gamma
IL-10: Interleukin-10
IL-6: Interleukin-6
IP-10: Interferon gamma-induced protein 10
MEDLINE: Medical Literature Analysis and Retrieval System Online
OIE: World Organisation for Animal Health
PCR: Polymerase chain reaction
PPE: Personal protective equipment
PRISMA: Preferred Reporting Items for Systematic Reviews and Meta-Analyses
PRNT: Plaque reduction neutralization test
RNA: Ribonucleic acid
RRR: Relative risk ratio
UK: United Kingdom
WHO: World Health Organization
WOAH: World Organisation for Animal Health
WoS: Web of Science
